# Trophic Interactions of European Hake *Merluccius merluccius* (Linnaeus, 1758) in Benthic Communities off the Moroccan Mediterranean Coastline: Seasonal and Ontogenetic Shifts

**DOI:** 10.1155/2023/8865128

**Published:** 2023-12-27

**Authors:** Douaa Slimani, Souad Abdellaoui, Najib El Ouamari, Nassir Kaddouri, Khaoula Kasmi, Rajae Mouedden, Mostafa Layachi, Jamal Settih, Khalid Chaabane

**Affiliations:** ^1^Laboratory for the Improvement of Agricultural Production, Biotechnology and the Environment, FSO, Mohammed First University, Oujda, Morocco; ^2^Regional Center of the National Institute of Fisheries Research, Fishing Laboratory, Casablanca, Morocco; ^3^Regional Center of the National Institute of Fisheries Research, Fishing Laboratory, Nador, Morocco; ^4^Department of Biology, Biodiversity, Ecology and Genomics Laboratory, Faculty of Sciences, Mohammed V University, Rabat, Morocco

## Abstract

The European hake, *Merluccius merluccius* (Linnaeus, 1758), is one of the most important resources for Mediterranean fisheries. Due to its pivotal role in energy transfer from lower to higher trophic levels, this species is a crucial component of the ecosystem's functioning. The ecological role of *Merluccius merluccius*, off the Moroccan Mediterranean Sea (southern Alboran Sea), was investigated, exploring seasonal and ontogenetic shifts, geographical variations in prey composition, and feeding strategy. Between November 2020 and July 2022, a total of 402 hake specimens were collected by oceanographic bottom trawl surveys (MEDITS) that were carried out during warm and cold seasons to assess their diet and feeding habits. The sample was analyzed according to fish sizes and seasons, and qualitative/quantitative feeding indices were calculated. The trophic spectrum of *Merluccius merluccius* included 24 prey items in total, mainly belonging to *Osteichthyes* (12), *Crustacea* (10), *Cephalopoda* (1), and *Polychaeta* (1), suggesting a generalist behavior of this predator as in numerous regions of the Mediterranean Sea, with several species that occasionally occurs in its diet. In the Moroccan Mediterranean Sea, *Osteichthyes* proved to be the most important prey item (%IRI = 78.56) among the different zoological groups, followed by *Crustacea* (%IRI = 16.22). The other food items were occasionally and randomly consumed, and cannibalism was low (0.8%). Hierarchical cluster analysis and nonmetric multidimensional scaling (nMDS) showed different feeding habits of two main groups separated at 60% similarity: small specimens <10 cm TL, primarily feed on zooplanktonic prey, while medium and large specimens hold a diet based on *Osteichthyes* with crustaceans. Furthermore, a significant positive relationship between hake and fish prey size was confirmed. Seasonally, mesopelagic *Osteichthyes* were the main food prey in the summer season, while pelagic species were predominant during the autumn. SIMPER analysis revealed that the prey items contributing the most to the differences between seasons and length classes were *Engraulis encrasicolus*, *Micromesistius poutassou*, *Boops boops*, *Macroramphosus scolopax*, gobids, *Gadiculus argenteus*, and most of *Crustacea*. The diet does not appear to be influenced by sex (>0.05). A trophic level (TROPH) of 4.1 was calculated, indicating that the species is a top predator (quaternary consumers). The TROPH values ranged between 2.58 and 4.38 from juveniles to adults, increasing asymptotically with the size of specimens. In contrast to what has previously been found in other Mediterranean regions, where ichthyophagous hake feed mostly on pelagic *Osteichthyes*, such as *Engraulis encrasicolus, Sardina pilchardus*, and *Micromesistius poutassou*, the study points up the vital role played by Atlantic horse mackerel *Trachurus trachurus* for hake diet in the Moroccan Mediterranean Sea. Information on the feeding ecology of fish species as provided in this study is essential to improve ecosystem conservation in accordance with multispecies approach to fishery management, leading to a better understanding of the role of hake in the Moroccan Mediterranean Sea demersal communities.

## 1. Introduction

The European hake, *Merluccius merluccius* (Linnaeus 1758), is an essential benthopelagic predator, naturally distributed in the continental shelf of the northeastern Atlantic Ocean, from Norway and Iceland to Mauritania including the Mediterranean Sea and the Black Sea, in depths between 30 and 1000 m. Higher abundance peaks of the species have been reported in depths ranging from 50 to 400 m [1]. This species represents an essential fishery resource for bottom trawls across its entire distribution and forms the basis of commercial demersal fisheries within the Mediterranean Sea ecosystem countries [2, 3], with an average catch over the period 2018–2020 of 18 945 t yr^−1^ for the Mediterranean Sea generating USD 177 million in terms of value according to FAO reports [4] and 8010 t yr^−1^ by Moroccan Mediterranean fisheries [5].

Indeed, its economic and social relevance, being the third most valuable species for the Mediterranean fisheries, has led to an overexploitation of several *M. merluccius* Mediterranean stocks, suffering from the highest fishing mortality among the demersal species focused on recruits and age group <1 [6], especially in the central and western Mediterranean Sea [7]. Thus, its management has become a growing concern in recent decades for the international communities, social parts, and scientists to identify proper operative management units (i.e., stocks). The assessment of hake in the Mediterranean Sea is undertaken annually by the Working Group on Stock Assessment of Demersal Species (WGSAD) of the General Fisheries Commission for the Mediterranean (GFCM) of the Food and Agriculture Organization of the United Nations (FAO). Several international institutions, commissions, and conventions have focused their efforts on the conservation status of *M. merluccius* (i.e., GFCM of the FAO, Scientific, Technical, and Economic Committee for Fisheries STECF, the International Council for the Exploration of the Sea ICES Working Groups, and the multiannual plan MAP in the EU).

In fact, from an ecological point of view, *M. merluccius* occupies a high level in the marine trophic web with a fundamental role as a relevant top predator in the trophic ecology of mesopelagic communities, maintaining the equilibrium and sustainability of the ecosystem and regulating the abundance and diversity of other species within their food web through consistent predation, ensuring a favorable impact on biodiversity and helping to preserve its natural state [8–10]. Therefore, to support the sustainability of this resource, management strategies must rely on accurate information regarding ecological and biological aspects based on objective data obtained through scientific studies of the populations subject to exploitation. Research on the trophic dynamics of marine organisms, especially of those playing a key role in their ecosystem, such as apical predators that occupy a high level in the marine trophic web, is essential to deepen the knowledge concerning ecosystem dynamics and functioning (i.e., trophic web) and energy transference between species in marine communities [8, 9, 11, 12]. Furthermore, information on interspecific biological interactions, prey preferences, and consumption rate in the different geographical areas is needed when conducting fish stock assessment to integrate prey-predator relationships in multispecies population dynamics, which allows scientists and managers to implement an ecosystem analysis based on food web models (i.e., Ecopath-Ecosim) to create an overall picture [13].

In the last decade, several efforts have been made by the scientific community to investigate the biology and ecology of *M. merluccius*. In the Mediterranean Sea, the juvenile hake diet was investigated using stomach content analysis [14, 15], while the feeding behavior of adult fishes was studied using carbon-nitrogen stable isotopes, stomach content analysis, and metabarcoding approach [16–19]. As reported by several authors [9, 10, 14, 20–23], *M. merluccius* feeds on a broad spectrum of prey including zooplankton, crustaceans, fish, and cephalopods, shifting its feeding habits geographically from one prey to another according to prey abundance and availability in the environment, described as an opportunistic feeder. Anchovies (*E. encrasicolus*) and sardines (*S. pilchardus*) are reported to be the main important prey both in the north-western Mediterranean [15], Adriatic Sea [24], and Tyrrhenian Sea [17], with high variability in the abundance of these small pelagic fish across subregions [10, 23]. In the Egyptian coastline (Eastern Mediterranean Sea [25]) and the Cantabrian Sea [8, 9, 26], blue whiting (*M. poutassou*) is dominant in hake diet, followed by anchovy and Atlantic horse mackerel (*T. trachurus*). In the northern Bay of Biscay [11, 27], the main fish prey for hake between 21 and 31 cm TL are anchovy, pilchard, and argentine (*Argentina sphyraena*). Hake larger than >32 cm TL feed mainly upon Atlantic horse mackerel.

Its diet also shows changes related to their ontogenetic development mainly appearing over 18 cm in TL, depending on the population [23]. In general, a planktivorous phase is reported in immature individuals (hake <15 cm TL indicated as size classes I and II in the present paper), mainly preying on Euphausiacea, Mysidacea and, to a lesser degree, on small benthic fishes [1, 17, 18, 23], while an ichthyophagous diet composed mainly by larger pelagic and nektobenthic fishes was reported for adults up to 20 cm of TL [1, 8, 15, 17, 20, 23, 24].

However, to date, there has been no detailed study on the trophic ecology of hake in the Moroccan Mediterranean Sea (MMS) in relation to the variation of its prey dynamics, which could help to better characterize the diet of the southwestern Mediterranean hake.

In the present case, we analyze for the first time in MMS the diet composition, feeding strategy, niche breadth, and trophic level of *M. merluccius*, considering seasonal and ontogenetic variability, through the analysis of stomach contents, to expand the knowledge on its ecological role and to evaluate the trophic relationships and trophic behavior of the species. This is essential both to improve available tools for the development of multispecies assessment models and to monitor the demersal and mesopelagic communities in one of the most heavily impacted Mediterranean geographical areas [6].

## 2. Materials and Methods

### 2.1. Study Site and Samples Collection

**Figure 1:**
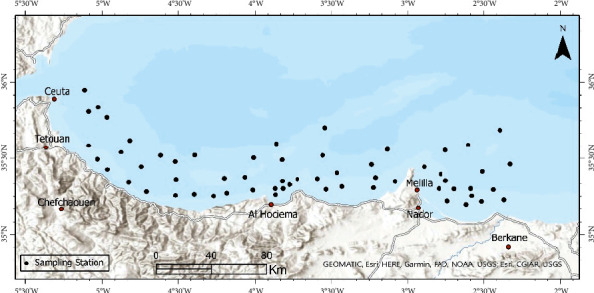
Map of southern Alboran Sea showing the location of sampling stations.

Experimental bottom trawl fishing was conducted in the Moroccan Mediterranean Sea (GSA 3 according to GFCM-FAO classification; Figure 1) within the framework of the MEDITS survey program (Mediterranean International Bottom Trawl Survey). Sampling was carried out with a scientific bottom trawl fishing boat in deep waters ranging between 20 and 620 m depths during two years (2020–2022).

During each survey, sixty daytime hauls of 30 to 60 minutes each were taken at an average speed of 3 knots, for a total of 240 hauls during the entire study period. In total, 402 *M. merluccius* were captured and 230 were examined for diet, with total length (TL) ranging from 7 to 54 cm.

On board the vessel, captured specimens were weighted (TW), measured (TL), sexed, and had their degree of sexual maturation determined according to [28]. After fish have been eviscerated, their stomachs were immediately preserved in ethanol 70% in order to interrupt the process of digestion until the contents could be studied. Afterwards, in the laboratory, each dietary item was identified under a binocular microscope to the lowest taxon level possible, then counted and weighed with 0.1 mg accuracy after removing excess water with blotting paper. When the state of digestion was more advanced, prey were determined to the level of genus or group and grouped into undetermined fish, crustaceans, and cephalopods.

### 2.2. Data Analyses

To investigate changes in feeding intensity during the growth of *M. merluccius*, the vacuity index (%VI) was calculated as VI=(Ne/*N*) *∗* 100, where *Ne* represents the proportion of empty stomachs while *N* refers to the total number of examined stomachs.

Three standard indices [29] were calculated to estimate the contribution of each prey to the diet: the frequency of prey occurrence (%*F*), the numeric prey composition (%*N*), and the weight composition (%*W*). These indices mentioned above were combined to calculate the Index of Relative Importance (IRI), an indicator of prey preferences, to get more precise results of diet as follows: IRI=(%*N*+%*W*) *∗* %*F* (proposed by Pinkas et al. [30] and modified by Hacunda [31]). Expressed as percentage %IRI=((IRI *i*)/(∑(IRI) ))*∗*100 [32, 33], it was used to perform statistical analysis on seasonal diet composition for each ontogenetic class.

Costellographical method with a two-dimensional representation of prey-specific abundance (Pi) and frequency of occurrence (*F*%) of the various prey (proposed by Amundsen et al. [34]) was used to determine feeding strategy (generalist or specialist) and prey importance (dominant or rare).

In mathematical terms,

(1) $$\begin{array}{c} \text{Pi}\%=\frac{\sum \text{stomach content }\left(\text{as volume},\text{weight},\text{or number}\right)\text{ comprised of prey }i}{\sum \text{stomach content in only those containing prey }i}\times 100. \end{array}$$

Prey importance rises along the diagonal from the lower left (insignificant prey) to the upper right corner (dominant prey). By analyzing the positions of the points on the vertical axis, we can gain insight into the feeding behavior adopted by the predator in terms of specialization or generalization.

In our pursuit of understanding the potential correlation between the abundance of prey species in the area and the dietary preferences of *M. merluccius*, we employed a targeted analytical approach. Specifically, we constructed a graph that juxtaposed the frequency of hake stomachs containing *T. trachurus* against the estimated catches of *T. trachurus* during the MEDITS surveys spanning from 2020 to 2022, focusing on hauls where hakes were captured. The selection of *T. trachurus* as the focus for our analysis stemmed from our prior observations of a little specialization tendency of *M. merluccius* towards this particular prey.

### 2.3. Statistical Analysis

A Pearson's chi-square test (*X*^*2*^) was applied to test for any significant differences in the frequency of empty stomachs among the different size classes.

Many factors affect the feeding habits of fish; consequently, a nonparametric Kruskal–Wallis ANOVA, followed by Tukey's test was performed first to qualify if the diet composition of the *M. merluccius* specimens varied statistically. Five factors were taken into consideration when designing the ANOVA: season (two levels: autumn and summer), sex (two levels: females and males), fish size (five levels detailed below), depth (0–100 m, 101–200 m, and 201–300 m), bottom nature of trawled areas (three levels: muddy, sandy, and hard), and trawling time (two levels: AM and PM).

To investigate possible variations in diet related to growth, samples were divided into five ontogenetic length classes based on published literature on hakes' biology [23, 35, 36]: class I (TL < 10 cm), class II (10.5–15 cm), class III (15.5–20 cm), class IV (20.5–32.5 cm), and class V (TL > 32.5 cm). For the classification and ordination of the different *M. merluccius* size groups with similar diets, Hierarchical Ascending Classification (HAC) and nonmetric multidimensional scaling (nMDS) were used, respectively. To better represent the trophic food web of different size classes, Gephi software (https://gephi.org) was used [37]. SIMPER analysis was performed on season, found to be significant in ANOVA, to evaluate the prey that were most responsible for the similarity/dissimilarity between and within groups in each season. The cluster analysis, nMDS, and SIMPER tests were performed using the statistical software PRIMER 6 and Past (V.4). *p* value was set at *p* < 0.05.

### 2.4. Ecological Indices

The Shannon–Wiener index (*H*′) was used to evaluate the diet diversity between size classes and seasons, based on prey taxa abundance. Statistical differences in the diet diversity were tested by analysis of variance (ANOVA).

Dietary overlap *Cλ* of *M. merluccius* between seasons, sexes, sexual maturity stages, and size classes was calculated using the Morisita–Horn index (*Cλ*; [38–40]). According to [41], a *Cλ* varied from 0 (low dietary overlap) (no items in common) to 1 (complete overlap). Trophic niche breadth was determined using Levins' index (Bi) [42]. Using the same values for *Cλ*, the niche breadth Bi was evaluated as follows: low values indicate a diet dominated by a few prey items (specialist predators), while higher values indicate generalist diets [43].

Trophic level was calculated to estimate the position of *M. merluccius,* using the following equation (2):

(2) $$\begin{array}{c} \text{TL}\kappa =1+\left(\overset{n=24}{\underset{n=1}{\sum }}\text{Pjx}*\text{ TLj }\right), \end{array}$$

where TL*κ* is the trophic level of the species of interest, *n* is the number of prey species, Pjx is the relative proportion of each prey in the predator's diet, and TLj is the fractional trophic level of each identified prey taken from FishBase (https://www.fishbase.org; [44]) or SeaLifeBase (https://www.sealifebase.org; [45]).

To test for any significant differences among sexes, sexual maturity stages, size classes, and seasons, a one-way analysis of variance (ANOVA; *F*-test) was applied.

### 2.5. Ethical Statement

The experiment procedures were assessed under the MEDITS program (Regulation (EU) 2017/1004) as part of annual research surveys. All applicable international, national, and/or institutional guidelines for the care and use of animals were followed by the authors.

Details regarding the sampling protocol and research methodologies can be found in the publication by Bertrand et al. [46].

## 3. Results

The results of a two-way ANOVA, performed on the abundance data of prey categories, revealed highly significant interaction effects for hake diet among length size classes and season, whereas no differences between sexes, depth, bottom nature, and trawl time were found (*p* > 0.05; Table 1). For this reason, all analyses were conducted with separate datasets.

### 3.1. Analysis of Feeding Dynamics

A total of 402 stomachs of *M. merluccius* were sampled for stomach content analysis (SCA); overall, 172 had completely empty stomachs, representing 42.7% of the total. The remaining 230 individuals were used to analyze the diet (57.3%), ranging between 7 and 54.5 cm TL.

While no significant differences were found in the frequency of empty stomachs by sexes (*χ*^2^ = 1.41; *p* > 0.05) and seasons (*χ*^2^ = 0.001; *p* > 0.05), differences were detected among the five size categories (*χ*^2^ = 15.86; *p* > 0.005). As shown in Table2, the vacuity index (VI) has increased in value from size classes I to IV, then decreased in the last size classes from IV to V, with a maximum of 28.5% and a minimum of 6.98%.

### 3.2. General Diet Description

A total of 230 stomachs with prey were used to analyze the diet of *M. merluccius*. 434 prey items were identified through the analysis of stomach contents, mainly belonging to 24 taxa of 4 major groups: *Osteichthyes*, *Crustacea*, *Cephalopoda*, and *Polychaeta* (Table 3). According to SCA results, among the principal prey categories, *Osteichthyes* (10 different taxa) emerged as the abundant group in the food composition according to all dietary indices, identified in higher abundance, weight, and occurrence, followed by *Crustacea*, while Euphausiacea and *Mysidacea*, *Cephalopoda*, and *Polychaeta* were found of lesser importance in stomach contents, and were absent in the diet of hakes larger than 32 cm TL (Tables 2 and 3). In particular, among *Osteichthyes* groups, pelagic *Osteichthyes* were the best represented taxa (in terms of IRI% = 56.8), followed by mesopelagic *Osteichthyes* (IRI% = 21.6) and *Osteichthyes* n.i. (IRI% = 5.1). *M. merluccius* were shown to be piscivorous that consume fishes as principal prey items. As shown in Table 3, for species level of the identified prey, Atlantic horse mackerel *Trachurus trachurus* (IRI% = 47.17) was the most representative species, followed by silvery lightfish *Maurolicus muelleri* (IRI% = 22.88), *Osteichthyes* n.i. (IRI% = 10.13), *Sardina pilchardus* (IRI% = 8.34), and *Myctophidae* (IRI% = 2.97). From *Crustacea* groups (IRI% = 13.52), shrimp *Parapenaeus longirostris* was the preferred prey category, occurring in 6% of all examined stomachs and constituting 6.22% by number and 2.22% by weight, followed by *Plesionika* sp., *Crustacea* n.i., *Processa* sp, *Pasiphaea sivado*, *Solenocera membranacea*, and *Nephrops norvegicus* comparatively in lower proportions in the stomach contents. Digested *Crustacea* occurred in 3.91% of all examined stomachs, its relative importance index was 0.67% (Table 3). Cannibalism was relatively rare in hake diet, being recorded in only 0.87% of stomach contents and 0.032% of IRI.

Qualitatively, the Shannon–Weaver index indicates in both sexes a similarity in the diversity of prey ingested (*H*′ = 2.4 for females; *H*′ = 2.3 for males). Quantitatively, females ingest twice as many voluminous prey as males (An♂ = 4.93, Aw♂ = 11.08 g; An♀ = 5.47, and Aw♀ = 26.06 g; Table 2). The Spearman correlation coefficient confirms the heterogeneity of the diet between the two sexes (*ρ* = 0.85, *t* = 7.39, and *α* < 0.05).

**Figure 2:**
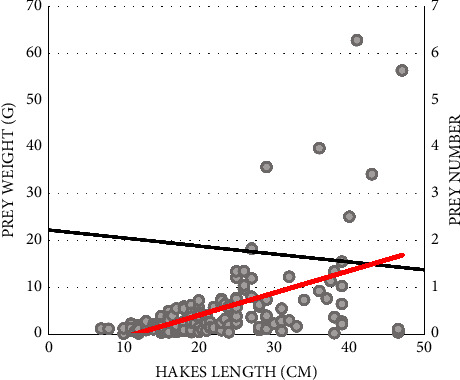
Relationship between hake length and prey weight (red line) and hake weight and number of prey species found in the stomach (black line).

According to Figure2, larger hake specimens tend to consume voluminous prey, and so there may be a positive correlation between predator weight and prey size. However, the number of prey consumed by a predator decreases with its growth.

### 3.3. Feeding Strategy

**Figure 3:**
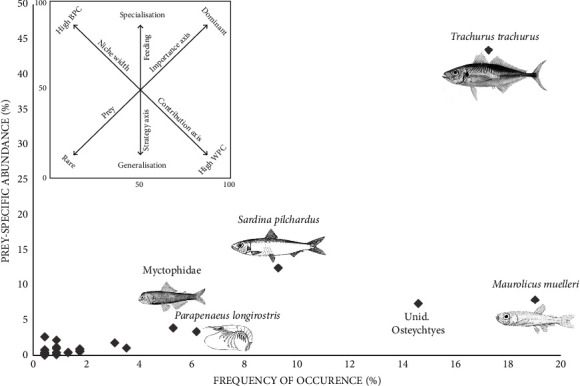
Relationship between prey-specific abundance (PSA) and frequency of occurrence (%*F*) of prey items in the diet of *Merluccius merluccius*, collected in the Moroccan Mediterranean Sea, including the explanatory diagram modified by Amundsen et al. (1996).

Feeding strategy plots (Figure 3) showed that *T. trachurus* can be defined as the dominant prey in *M. merluccius* diet in the study area (the highest value of PSA obtained was 0.4). Therefore, some individuals within the population had a strategy of specialization towards the pelagic *Osteichthyes* category. Conversely, most prey species are located close to the *x*-axis in the lower left corner of the diagrams, a region of low prey importance at the population level being consumed by a low percentage of predators and rarely seen. This suggests that *M. merluccius* in the Moroccan Mediterranean Sea held a somewhat generalist niche with a low specialization for pelagic *Osteichthyes*. Regardless of the number of prey items in their diet, these predators tended to prefer five particular prey species: *T. trachurus*., *M. muelleri*, *S. pilchardus*, and *Myctophidae*, while *P. longirostris* was the most important crustacean item.

**Figure 4:**
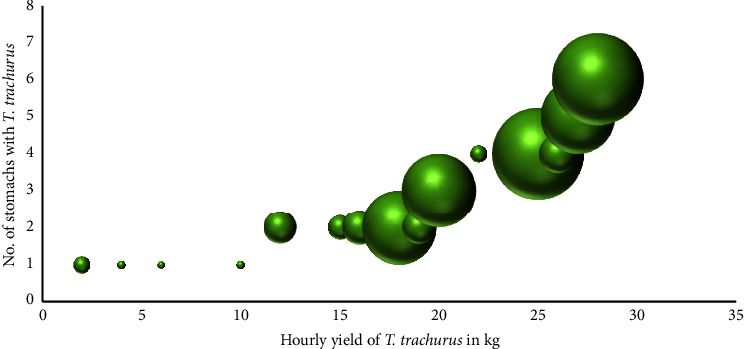
Prey-predator functional relationships. Number of *Merluccius merluccius* stomachs containing *Trachurus trachurus* in relation to the biomass of *Trachurus trachurus* estimated for the same hauls (data from MEDITS 2020–2022 surveys). The bubble size is proportional to the number of *Merluccius merluccius* stomach data (number of individuals) available per haul.

Notably, when plotting the number of *M. merluccius* stomachs containing *T. trachurus* in relation to its catches estimated for the same hauls obtained from the MEDITS 2020–2022 survey (Figure 4), we found a strong correlation between its presence in the diet and its catches in the sampling area, confirming the opportunistic feeding strategy of *M. merluccius*.

### 3.4. Ontogenetic Changes in Diet

**Figure 5:**
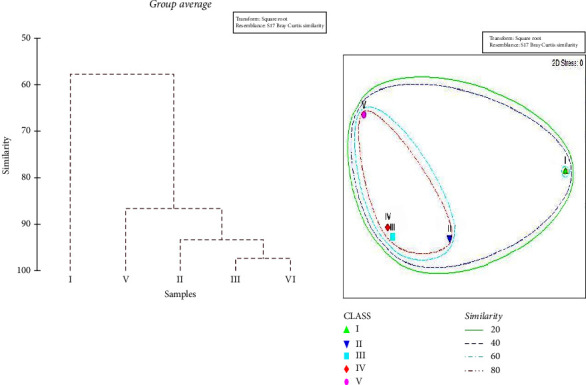
Dendrogram and nMDS ordination of Bray–Curtis similarities from dietary data (square root transformation) for the five hake ontogenetic stages analyzed. Cluster A included only the specimens belonging to class (I), while cluster B included the specimens belonging to classes II–V.

Cluster analysis and nMDS ordination based on the IRI showed clear diet variation from the smallest juveniles to adults pointing out two main clusters (Figure 5). Cluster A included only the specimens belonging to class I, while cluster B included the specimens belonging to classes II, III, IV, and V with similar values of %IRI.

**Figure 6:**
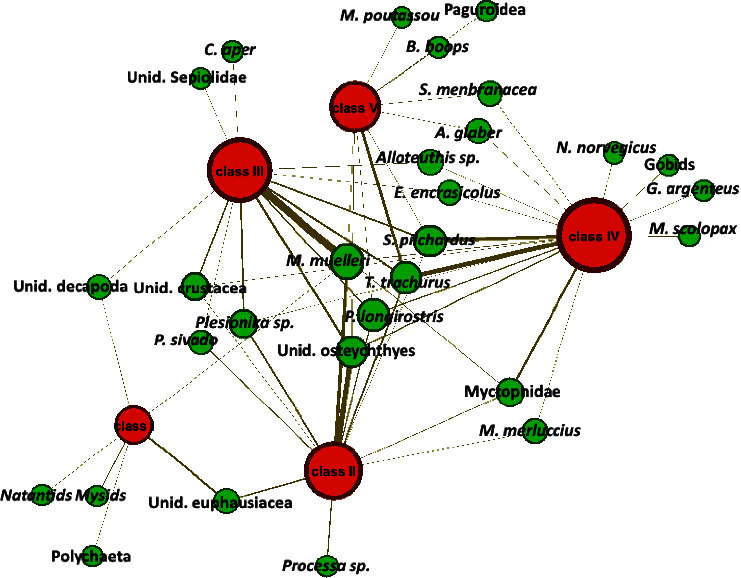
The food web of the different size classes of *Merluccius merluccius*, with red nodes representing the predator divided into five different size categories and green nodes signifying the prey. The nodes' size is proportional to the number of links connected, while the size of links is proportional to the number of times the prey-predator relationship was detected in the samples. The species are arranged based on their relationship with the predator's size categories, with the prey species shared by all size categories situated in the center.

**Figure 7:**
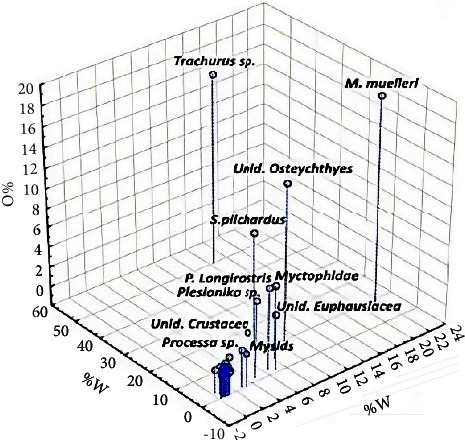
Three-dimensional representation graph of hake's diet *Merluccius merluccius* described by the numerical, gravimetric, and frequency of occurrence.

As reported in Table 2 and Figure 6, the smallest individuals (size class I; TL < 10 cm) primarily fed on *Euphausiacea* and *Mysidacea* (IRI% = 98.17), followed by mesopelagic *Osteichthyes* (IRI% = 1.24) and decapods with crustaceans (IRI% = 0.57). Class II (10.5 < TL < 15 cm) presented a diet where the *Euphausiacea* and *Mysidacea* were still current prey, but the hakes' preferences were significantly shifted towards larger decapod and crustacean prey (IRI% = 34.43), such as *P. longirostris*, and teleost fishes with the highest %*N* and %*O*, such as *T. trachurus* and *M. muelleri* (Figures 6 and 7). As hake grows, we have noticed that the importance of fish strongly increases further in the diet composition, while a simultaneous decline in consumption of crustaceans was noted. The importance of demersal and pelagic *Osteichthyes* was well evident by the highest IRI% (39.98) in the diet of the size class III (TL between 15.5 and 20 cm), followed by mesopelagic *Osteichthyes* (32.67), decapods and crustaceans (16.07), and *Osteichthyes* n.i. (8.31). From the size group IV (TL between 20.5 and 32.5 cm), a shift towards larger demersal prey such as blue whiting (*M. poutassou*) and a simultaneous decline in the proportion of mesopelagic *Osteichthyes* were noted. Demersal and pelagic *Osteichthyes* account for the most frequent prey with a high value of IRI% (90.4). The teleost fishes represented almost the entire diet at size group V (TL > 32.5 cm), as confirmed by the highest IRI% values of different *Osteichthyes*, where they became the dominant prey group (IRI% = 98.5). The niche breadth index Bi decreases during ontogenesis, indicating a tendency towards less generalized feeding as hake grows.

The one-way ANOVA was performed to assess dietary composition differences among size classes of the *M. merluccius* specimens. Indeed, significant dietary differentiations were found between class I from all other size classes (*p* < 0.05), while the other classes did not differ significantly in their diets (*p* > 0.05). In detail, classes II, III, IV, and V showed the 60% of similarity mainly possessed by the large contribution of *Osteichthyes* (Figure 5 and Table 2). The food web network permitted us to recognize the number of prey that were shared among the different size classes (Figure 6). In particular, the web of trophic interactions generated through the SCA approach revealed that *M. muelleri*, *T. trachurus*, *S. pilchardus*, and *P. longirostris* were the species that were shared among all size classes. In addition, the network revealed the presence of clouds of size-specific prey species that were exclusively found in one size class, such as *M. poutassou* and *B. boops*, found to be preferred by larger *M. merluccius* individuals parallel *Natantids*, *Mysidacea*, and *Euphausiacea*, consumed by juveniles, demonstrating a highly complex trophic interaction system of European hake in the Moroccan Mediterranean Sea.

### 3.5. Seasonal Changes in Diet

Diet composition was significantly different between the two sampling seasons (*p* < 0.05). Analysis of IRI (Table 2) showed that pelagic and mesopelagic *Osteichthyes* with crustaceans were present in the diet of this species in both seasons. Specifically, during the summer months, the diet included 16 prey items (7 fishes and 9 invertebrates). According to the %IRI, *M. merluccius* fed mainly on mesopelagic *Osteichthyes*, represented in the diet by *Maurolicus muelleri* and myctophids, then replaced by pelagic *Osteichthyes*, becoming an important food item for *M. merluccius* in autumn, represented mainly by *Trachurus trachurus* and *Sardina pilchardus*. The diet included 14 prey items (11 fishes and 3 invertebrates) in this period. Overall, more families of demersal *Osteichthyes* showed maximal value in the stomachs of individuals caught in the cold season than those caught in the warm season. Decapods and crustaceans were present in the diet throughout the year, with a peak in summer (%IRI = 16.28). All the other prey items were classified as accidental. Cephalopods were present in stomach contents during autumn but in lower quantities. SIMPER analysis showed high dissimilarity (65.22%) and identified those prey categories that showed a clear separation to European hake diet between the two seasons (Table 4). An increased fish content was observed in autumn (*Engraulis encrasicolus*, *Micromesistius poutassou*, *Boops boops*, *Macroramphosus scolopax*, gobids, and *Gadiculus argenteus*), while most crustaceans such as *Nephrops norvegicus*, *Pasiphaea sivado*, *Plesionika* sp., and *Solenocera membranacea* were only found in summer. *Euphausiacea* and *Mysidacea* were identified in the stomachs of individuals caught in autumn, whereas *Polychaeta* were consumed only by individuals caught in summer. Cannibalism phenomena were also identified in the guts of *M. merluccius*, occurring equally in both seasons (Table 4).

### 3.6. Study of Trophic Relationships

#### 3.6.1. Trophic Niche Breadth and Diet Overlap

According to the classification of niche breadth size, *Bi* was found to gradually decrease with an increase in the total length of predators (Table 2). Indeed, small individuals (size I) were found to be generalist feeders (Bi = 0.71), whereas adults indicate Bi values lower than 0.6 (more specialization).

Morisita–Horn index values indicated high overlap between all categories of hake, with values close to 1, confirming the similarity in their diets described above. The diets of females and males were practically identical (*Cλ* = 0.98), i.e., there was an almost total overlap between them. The same happened between the two maturity classes (<24 cm and >24 cm TL). Following this trend, the immature and mature females had highly similar diets, and this was the same case between immature and mature males (Table 5).

#### 3.6.2. Trophic Position and Ecological Role of *Merluccius merluccius* in the Moroccan Mediterranean Waters

The trophic level calculated for the total population was 4.1 units, which suggests that hake could be considered carnivorous with a preference for fishes and decapods (2.2 < TL < 4.35). The TROPH values calculated related to sex were almost the same: 4.3 for males and 4.2 for females. Juveniles (TL < 24 cm) had a trophic equal to 3 and 4.4 for mature ones (TL > 24 cm). The highest estimated trophic level was for mature females (4.5) and the lowest for immature individuals (class I: 2.58). The TROPH of hake did not exhibit significant changes with the season, recording 4.08 in autumn and 4.13 in summer (*p* > 0.05; df = 1; Table 6).

## 4. Discussion

### 4.1. Overall Diet Composition and Feeding Habits

European hake is a highly important species for fisheries, particularly in Moroccan Mediterranean waters, where it accounts for a significant proportion of the demersal catch, making up to 10% of landings [5]. However, like many other commercially important fish species, it is recommended that European hake in this area to be managed carefully, as it has been rated as overexploited [47].

This study provides the first comprehensive analysis of hake diet preferences and feeding habits from the entire area of the Moroccan Mediterranean Sea (MMS; GSA3).

Our results revealed a decreasing feeding rate with increasing length class of specimens, as can be inferred from the percentage of empty stomachs, with lower values recorded in young individuals, which agrees with previous studies from the Mediterranean Sea [23, 36]. Conversely, El habouz et al. [48] reported higher %VI values in *M. merluccius* from the Atlantic Moroccan waters, especially in large specimens, thus indicating an increasing feeding intensity with growth, suggesting that larger specimens were more experienced feeders. Such fluctuations could be considered as a consequence of technique of sampling, namely, fishing gear, spawning activity, and everted and regurgitated stomach rates [49].

In line with literature obtained by different authors from other geographical areas [1, 8, 21, 22, 50], our data confirmed its ecological role as a generalist carnivorous predator in the MMS, feeding on a broad spectrum of benthic prey consisting predominantly of *Osteichthyes* and crustaceans, carrying this predation in different trophic levels. Regarding the high value of the trophic level (TROPH = 4.1), we confirm that *M. merluccius* occupies an important role as a carnivorous top predator in the trophodynamics of the fish community. Similar trophic values have previously been reported to *M. merluccius* in the Mediterranean basin (TROPH = 4.3 [17]).

The occurrence of species profoundly connected with the benthic environment in the stomach contents (*P. longirostris*, *A. glaber*, and gobid fish) as well as mesopelagic bioluminescent species (e.g., *Myctophidae*, *C. maderensis*, and *M. muelleri*), along with nektobenthic species (e.g., *T. trachurus*, *M. scolopax*, and *P. sivado)*, suggests that *M. merluccius* perform large horizontal and daily vertical migrations from the entire water column at night, to suprabenthic layer during daylight [1, 8, 10, 17, 21, 51]. Overall, these teleosts are an essential component of marine energy exchanges, linking zooplankton from lower trophic levels to top predators, creating a complex food web between pelagic and suprabenthic strata [16–18, 36, 52, 53].

In addition, research has consistently reported that hake's feeding habits shift geographically from one prey to another as a function of prey availability and abundance in the environment [9, 20, 21, 50, 54]. Therefore, *M. merluccius* diet reflects an opportunistic feeding scenario. The main difference in spatial distribution is related to the dominant pelagic fish preyed on by hake: *Trachurus trachurus* along with *Micromesistius poutassou* in the Cantabrian Sea [8, 9, 26], *Engraulis encrasicolus* and *Sardina pilchardus* in the north-western Mediterranean Sea, Adriatic Sea, and Tyrrhenian Sea [1, 15, 17, 24], and *Trachurus trachurus* and *Engraulis encrasicolus* in the northern Bay of Biscay [11, 27].

Arrestingly, the diet of hake in the MMS is more comparable with the species diet in the NE Atlantic than in the diet of hake in other sectors of the Mediterranean Sea [9, 11, 14, 55, 56], with *T. trachurus* being the dominant pelagic fish preyed on, followed by *M. muelleri*, *Osteichthyes* n.i., and *S. pilchardus.*

The Atlantic horse mackerel is a very abundant species in the MMS as documented by trawl and acoustic surveys [57]. The fact that *T. trachurus* is a key prey for hake in this area compared to other areas in the Mediterranean Sea probably is related to a local greater availability in the whole pelagic ecosystem in the MMS. This feeding strategy allows this species to take advantage of seasonal variations in food availability [58] maximizing their chances of survival in overexploited ecosystems.

In addition, distinguished from other geographical areas [18, 19, 23], cephalopods are negligible prey along the MMS (same as the finding of Bozzano et al. [8] and Carpentieri et al. [1]), even though they are considered abundant in the population of the area, notably the species octopus, squid, and cuttlefish [59–61]. This may be mostly due to the biological characteristics of communities in the area, which include a high prevalence of pelagic, benthopelagic, and mesopelagic fishes, which may be favored to hake as prey over cephalopods.

Furthermore, it has been commonly observed that hake consumes conspecific individuals [10, 11, 14, 50, 62–65]. However, in this study, cannibalism has been confirmed in hake diet, being recorded in only 0.8% of stomach contents, which is consistent with previous findings [8, 9, 23, 24, 48]. In other areas, cannibalism has been reported to exceed 12% of the total population (as noted by the authors of [1, 27, 66]).

### 4.2. Ontogenetic and Seasonal Variations in Diet Composition

From the numerical and biomass values, several studies observed that according to size with differences in the proportions of prey consumed, prey weight was proportional to the growth, which implies that fish, as it matures, it consumes more voluminous prey compared to smaller individuals [10, 67]. Interestingly, this is consistent with the observed pattern in the MMS, where a decrease in the number of prey with fish size was noted, and then an increase in their mass with fish size was noted. This suggests that larger fish tend to focus on consuming larger profitable prey items that provide a greater energy return. As for sex, our study shows that females of hake have a higher degree of voracity compared to males, consuming more voluminous prey. This can be attributed to various factors, including differences in the size as well as differences in their reproductive strategies since female individuals require higher energy and nutrients to support the development of their eggs and the production of offspring. As a result, they may consume larger or more nutrient-dense prey items to meet these increased nutritional demands. In addition, female fish may be more selective in their feeding habits, choosing prey that provides the most benefits in terms of energy and nutrition [68]. When it comes to diet shifts according to maturity stages, adults were found to have a piscivorous regime, preying mostly on fast-moving pelagic *Osteichthyes* which they ambush in the water column, linked to a specialization strategy for teleosts according to previous findings [1, 8]. .In the MMS, as well as in other nearby areas such as northern Sicily and the Atlantic coeasts, the largest specimens (≥45 cm) exhibited an exclusive piscivorous feeding behavior. This change in diet with predator ontogeny has been noted in other cogeneric species of the genus Merluccius as well (as stated in papers by Roel and Macpherson [62], Álamo and Espinoza [69], Iitembu et al. [70], and Belleggia et al. [71]). The observed changes in the diet composition after the first year of hake life indicate the adaptability of this species and can be attributed to multiple factors. Different authors [9, 19, 27, 72] suggested that size-related shifts to different diets could be linked to increased metabolic demands and genetic needs. In fact, as hake grows, larger individuals may change their nutritional requirements, requesting more food to sustain their energy needs; however, it does not explain our observations in European hake, as euphausiids show a high caloric content in comparison to fish [73–75]. Others have attributed these changes to morphological characteristics differences between juvenile and adult specimens [76, 77]. As hake grows, its ability to move through water and its speed increase, enabling larger individuals to explore more extensive areas for food and to pursue prey with greater speed and flexibility. In addition, according to [36], consuming one or another prey from different sizes is mainly related to mouth diameter. Having larger oral aperture allows adults to capture larger and more challenging prey (e.g., crabs and fish) that smaller ones cannot consume, with smaller cavity allowing them only to consume small-sized prey such as small crustaceans.

Seasonal variability in the diet of *M. merluccius* was also reported by Philips [25] for the Egyptian Mediterranean waters and Velasco and Olaso [9] for the Cantabrian Sea. In our case, prey diversity in the diet appeared to be fairly similar in both seasons in correspondence to specific prey occurrence (*H*′ = 2.4 and 2.1 in autumn and summer seasons, respectively). During the whole year, *Osteichthyes*, especially pelagic and mesopelagic *Osteichthyes*, are by far the dominant food item, but differences can be noted on other important items. Indeed, in accordance with earlier studies [8, 78], the main prey species in the diet such as *T. trachurus* and *S. pilchardus* were found to be the same all year round, especially in autumn. Diet composition may be influenced by the type and abundance of prey in their environment. Thus, seasonal changes in the availability of prey can significantly impact the diet of *M. merluccius*. The increase of ingested pelagic *Osteichthyes* in the autumn season could be explained by fish movements to shallow parts for reproduction, since the spawning season estimated for *M. merluccius* in the eastern, central, and western Mediterranean Sea [28, 79] and the eastern central Atlantic Moroccan coast [48] occurred in the autumn season from November to March.

For instance, the contribution of mesopelagic *Osteichthyes* was highest in the stomachs collected from the summer period, while shrimps were prey of secondary importance. This result is probably due to the high abundance of *M. muelleri*, *Myctophidae*, and shrimps in summer months in the MMS, which have a reproductive cycle taking place from March to September with a peak in spring [80, 81]. Similarly, as a result of a recent reproductive event of those prey items, their high-frequency occurrence index value in our samples could be associated with a peak of the abundance of those prey items in the area. In the Mediterranean Sea, mesopelagic fish represent an important trophic resource not only for hake but also for other teleosts (*Thunnus thynnus* and *Thunnus alalunga*) and dolphins [82–84]. This serves as evidence of the reverse transfer of energy from the deeper mesopelagic to the shallower epipelagic communities.

### 4.3. The Importance of Ecosystem-Based Fisheries Management: A Comprehensive View

Marine ecosystems are a complex system of interdependent trophic interactions including predation, nutrient cycling and energy transfer, between benthic and pelagic sectors, playing a critical role in connecting these habitats and supporting the functioning of aquatic ecosystems. Therefore, exploring the feeding habits and prey preferences of benthopelagic predators provides valuable insights into the complex predator-prey interactions, deepening the knowledge of species' role in the food web. Indeed, by delving deeper into the specific diet of voracious predators, such as European hake, researchers can better understand the functioning and dynamics of the marine ecosystem.

As active predators, hakes are flexible in their feeding behavior and able to migrate between different trophic levels, as shown by the results, representing a vital component in linking marine ecosystems, leading to a better understanding of trophic interactions in a global ecosystemic perspective approachable. Besides, they occupy a unique niche in the environment, acting as both predators and prey to different species, making them particularly sensitive to changes in their ecosystems, such as alterations in the abundance of their prey or alterations in oceanographic conditions. Therefore, they can function as crucial biological indicators of aquatic communities' health. Consequently, any kind of disturbance can have serious consequences for the stability of marine ecosystems, leading to imbalances within the predator-prey interactions. Indeed, understanding the interplay between fisheries and marine ecosystems is crucial in order to comprehensively evaluate the impacts that different fishing practices have on the environment. This is particularly relevant when it comes to mixed and heterogeneous fisheries operating in oligotrophic environments like the southern Alboran Sea. A deep understanding of these interactions is essential to analyze both direct and indirect effects of fisheries on marine ecosystems. By taking a complete perspective, researchers can identify the complex relationships between different species and better predict the potential consequences of fishing activities on the marine environment. Ultimately, this knowledge can inform more sustainable management practices that balance the needs of the fishing industry with the health and conservation of marine ecosystems.

Hence, the long-term sustainability of fisheries depends on maintaining the strength of the ecosystem by taking into account the entire ecological system rather than just focusing on individual fish species when managing fisheries. This approach considers the interactions between all living organisms in the ecosystem, environmental conditions, interactions with other species, and human activities.

## 5. Conclusion

The current study aims to increase the knowledge about *M. merluccius* in the MMS, confirms previous findings on the feeding habits of this species, characterizing it as an opportunistic top predator, feeding mainly on fish in all size groups and crustaceans. This bony fish displayed a diverse diet that did not change with sexes but varied according to fish sizes and seasons. The size-related shifts in food item preferences of *M. merluccius* appear to be influenced by their physiological requirements, while the seasonal variations in their dietary pattern could be a result of their opportunistic feeding behavior.

More comprehensive studies are needed using relatively recent techniques, such as stable isotope analysis, which has previously been applied to hake trophic ecology in the Adriatic Sea [18], and metabarcoding methods based on COI PCR amplification, which have so far, only been employed by Riccioni et al. [19] in the Strait of Sicily.

The results of this study can be integrated with biological and ecological data to develop future fishery assessment models of this species, providing crucial insights into the possible trophic cascade effects of the applied management measures towards more sustainable management actions of the relevant ecological and commercial fish communities and stocks and consequently the preservation of the entire marine ecosystem.

## Figures and Tables

**Table 1:** Statistical results of two-way ANOVA test applied for abundance data for *Merluccius merluccius* diet in the deep waters of the Moroccan Mediterranean Sea: comparison among seasons and total length classes (LT).

Source of variation	Degrees of freedom	Sum of squares	Mean squares	*F*	Pr > *F*	*p* value
SEASON	1	16,679	16,679	4,502	0.035	0.01 < *p* < 0.05
LT	4	40,454	10,113	2,814	0.026	0.01 < *p* < 0.05

**Table 2:** Diet composition in the five hake size classes sampled in 2020–2022.

	Length classes					Season	
	I	II	III	IV	V	Autumn	Summer
Vacuity index (%VI)	6.98	20.35	23.8	28.5	20.4	43.3	43.1
Bi	0.71	0.45	0.31	0.33	0.31	0.29	0.26
*H*′	1.65	2.17	2.19	2.22	1.82	2.4	2.1
*Cephalopoda*	—	—	0.49	0.04	—	0.22	—
*Crustacea*	0.23	35.11	18.27	3.65	7.08	6.97	16.28
*Euphausiacea* and *Mysidacea*	98.96	2.89	0.00	—	—	1.12	0.07
Demersal *Osteichthyes*	—	0.14	0.04	0.65	4.42	4.05	—
Pelagic *Osteichthyes*	—	6.87	19.00	88.22	86.45	0.51	67.53
Mesopelagic *Osteichthyes*	0.48	28.30	59.24	6.24	0.24	1.13	10.01
Unid. *Osteichthyes*	—	26.69	2.96	1.20	1.81	86.01	6.08
*Polychaeta*	0.33	—	—	—	—	—	0.03

In the column are reported the vacuity index (%VI), Levin's dietary niche breadth index Bi, and the Shannon–Weaver index *H*′ and IRI% values for each prey category.

**Table 3:** The general diet composition of *Merluccius merluccius* sampled in the Moroccan Mediterranean waters.

Prey category	%*O*	%*N*	%*W*	%IRI
*Cephalopoda IRI% = 0.08*				
*Alloteuthis* sp.	1.30	0.69	0.47	0.07
Unid. *Sepiolidae*	0.43	0.69	0.27	0.02

*Crustacea IRI% = 13.52*				
*Alpheus glaber*	0.87	0.46	0.52	0.04
*Paguroidea*	0.43	0.23	0.03	0.01
*Nephrops norvegicus*	0.43	0.23	0.24	0.01
*Parapenaeus longirostris*	6.09	6.22	2.23	2.25
*Pasiphaea sivado*	1.74	1.15	0.64	0.14
*Plesionika* sp.	5.65	4.61	1.58	1.53
*Processa* sp.	1.74	2.53	0.75	0.25
*Solenocera membranacea*	0.87	0.92	0.33	0.05
Unid. *Decapoda*	0.87	0.69	0.03	0.03
Unid. *Crustacea*	3.04	3.46	1.36	0.64

*Euphausiids and Mysidacea IRI% = 2.72*				
*Mysids*	1.30	3.00	0.36	0.19
*Natantids*	0.43	1.15	0.12	0.02
Unid. Euphausiacea	5.22	11.06	1.95	2.97

*Demersal Osteichthyes IRI% = 0.16*				
*Boops boops*	0.87	0.46	1.56	0.08
*Capros aper*	0.43	0.23	0.15	0.01
*Gadiculus argenteus*	0.43	0.23	0.18	0.01
Gobids	0.43	0.23	0.31	0.01
*Macroramphosus scolopax*	0.43	0.23	0.45	0.01
*Merluccius merluccius*	0.87	0.46	0.37	0.03
*Micromesistius poutassou*	0.43	0.23	2.83	0.06

*Pelagic Osteichthyes IRI% = 56.8*				
*Engraulis encrasicolus*	0.87	0.69	1.08	0.07
*Sardina pilchardus*	9.13	7.83	13.03	8.34
*Trachurus trachurus*	16.96	13.82	49.68	47.17

*Mesopelagic Osteichthyes IRI% = 21.6*				
*Maurolicus muelleri*	18.70	20.28	7.66	22.88
*Myctophidae*	5.22	7.83	5.17	2.97

*Osteichthyes n.i. IRI% = 5.1*				
Unid. *Osteichthyes*	14.35	9.45	6.68	10.14

*Polychaeta IRI% < 0.009*				
Polychaeta n.i.	0.43	0.92	0.00	0.02

For each prey item, the values of the following indices are shown: %*O* (frequency of occurrence), %*N* (percentage of number), %*W* (percentage in biomass), and %IRI (index of relative importance).

**Table 4:** SIMPER analysis showing species ranked according to average Bray–Curtis dissimilarity between the two seasons.

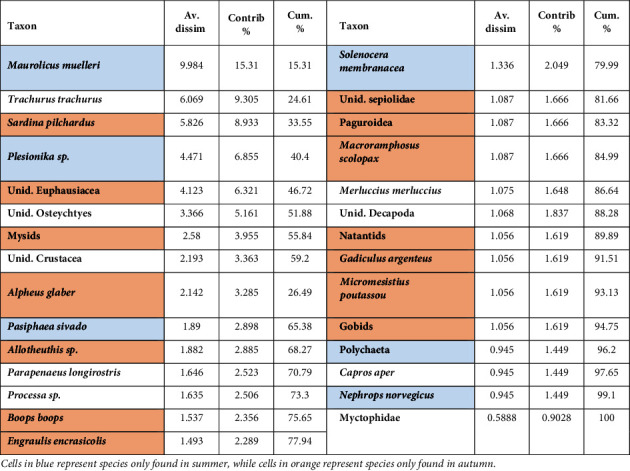

**Table 5:** Morisita–Horn index values of overlap among the different categories of hake.

Category	Overlap
Females vs males	0.98
Mature females vs immature females	0.99
Mature males vs immature males	0.88
Specimens of <24 cm and >24 cm	0.99

**Table 6:** The trophic level (TROPH) of each hake category.

Category	TL
General	4.1
Females	4.3
Males	4.28
Immature females	4.2
Mature females	4.5
Immature males	4.2
Mature males	4.3
Summer season	4.13
Specimens of <24 cm	3.02
Specimens of >24 cm	4.4
I	2.58
II	4
III	4.1
IV	4.35
V	4.38
Autumn season	4.08

## Data Availability

The datasets used and/or analyzed during the current study are available from the corresponding author upon reasonable request.
